# Detection of partial loss of hippocampal striation at 1.5 Tesla magnetic resonance imaging

**DOI:** 10.1186/s13244-019-0783-x

**Published:** 2019-10-26

**Authors:** Anitha Sen, Sudhakaran Sankaran

**Affiliations:** 1 Department of Radiodiagnosis, RCC, Thiruvananthapuram, 695011 India; 2 Department of Radiodiagnosis, Government Medical College Kottayam, Kerala, 686008 India

**Keywords:** Epilepsy, Hippocampal striations, Magnetic resonance imaging, 1.5 Tesla

## Abstract

**Objectives:**

Partial loss of hippocampal striation (PLHS) is recently described in 3 T and 7 T MR imaging as a sensitive indicator of hippocampal sclerosis. *Primary objective*: We described the demographic characteristics of the population with seizure disorder having PLHS at 1.5 T MR imaging and tried to see the relation of PLHS to the classic signs of hippocampal sclerosis. *Secondary objective*: PLHS was also looked for in a small control population that had no seizure history.

**Methods:**

This retrospective study had the approval of the institutional review board. In patients demonstrating PLHS on oblique coronal T2-weighted images, the following were recorded: age, sex, EEG findings, side of PLHS, hippocampal atrophy and high signal intensity of the hippocampus. In control population, the following were recorded: age, sex, presence/absence of PLHS and indication for imaging.

**Results:**

The 116 PLHS subjects (age range 2–73 years) included 62 males and 54 females. Sixty-six (56.9%) of our PLHS subjects were less than 18 years of age: 44 (37.9%) under the age of 12 years and 22 (19%) of 12–18 years of age. Classic signs of hippocampal sclerosis were found in only 7 (6%) of the 116 subjects showing PLHS. All patients with classic signs showed PLHS on the same side. Of the control population (25 subjects, age range 3–76 years, 17 males and 8 females), one showed PLHS—he was a treated case of CNS lymphoma with gliotic changes, though there was no history of seizure.

**Conclusion:**

PLHS is demonstrated at 1.5 T in both adult and paediatric population in this article and is much more common than the classic signs of hippocampal sclerosis (increased signal intensity and volume loss).

## Key points

- Partial loss of hippocampal striation (PLHS) (previously described in 3 T and 7 T) is demonstrated at 1.5 T in this study.
- PLHS (previously described in adults) is noted in both adult and children in this study.
- PLHS may be an easy technique for early detection of hippocampal sclerosis**.**

## Introduction

Epilepsy is prevalent in ~ 0.6% of the population. Temporal lobe epilepsy (TLE) is known to be the most common cause of focal epilepsy; 70% of TLE is associated with hippocampal (mesial temporal) sclerosis—which on pathology is neuronal loss and gliosis. TLE is often refractory to medicines, but ~ 60–70% of patients achieve long term seizure-free status [1] after resection of medial temporal lobe and varying amounts of temporal neocortex. Surgical candidates are identified based on EEG, MRI, PET/SPECT and neuropsychological tests. Identification of medial temporal sclerosis (MTS) or a structural lesion in medial temporal lobe by imaging is associated with a good surgical outcome [2].

The human cortex consists of neocortex (cerebral cortex) and allocortex (olfactory cortex and hippocampal formation). The hippocampal formation consists of dentate gyrus and surrounding hippocampus proper (cornu ammonis (CA) which is divided into four parts: CA1–CA4). Hippocampal sclerosis is best seen on MRI using thin coronal sections on T2-weighted or T2 fluid-attenuated inversion recovery (FLAIR) sequences. Classical (primary) signs of hippocampal sclerosis are atrophy and high signal intensity confined to the hippocampus. Secondary MR features are volume loss of temporal lobe, dilatation of choroidal fissure, narrowing of collateral white matter, forniceal asymmetry and atrophic mamillary body. MRI-negative TLE continues to be a significant problem.

Partial loss of hippocampal striation (PLHS) was recently proposed as a highly sensitive feature of hippocampal sclerosis on 3 T [3, 4] and on 7 T [5] MR. Since 1.5 T MR is more widely available, it would be very useful if this subtle finding could be consistently identified at 1.5 T. We tried to describe the demographic characteristics of the population having PLHS at 1.5 T MR imaging and to see the relation of PLHS to the classic signs of hippocampal sclerosis (increased signal intensity and volume loss).

## Materials and methods

### Patients and subjects

This retrospective study was approved by the institutional review board, which did not require informed consent.

**Fig. 1:**
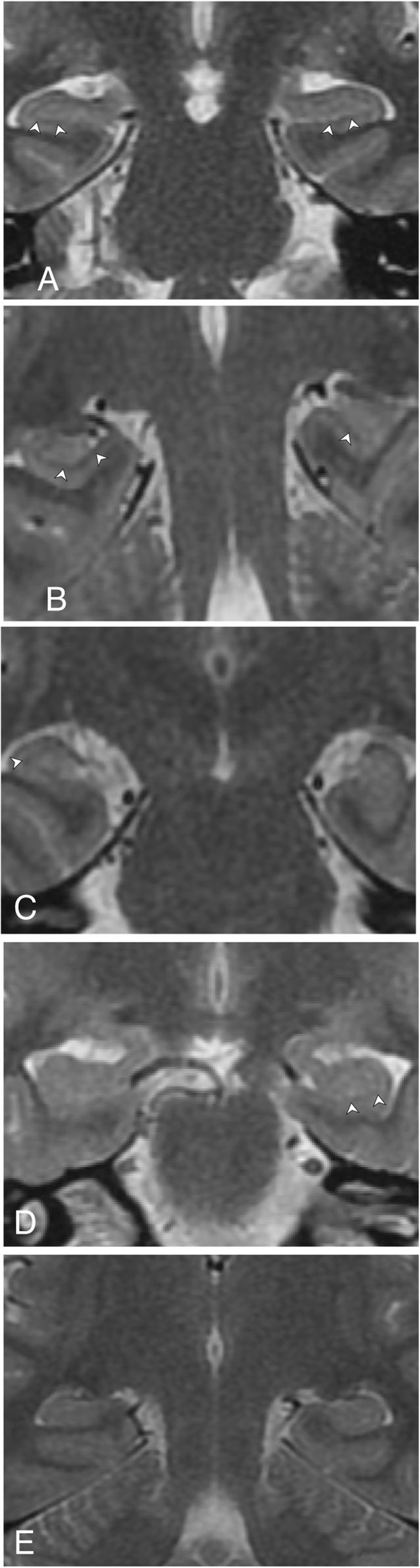
**a**–**e** High-resolution coronal T2-weighted images with magnification. Arrowheads indicate hippocampal striations. **a** Normal hippocampus: hippocampal striations seen on both right and left sides. Symmetry of semicircular canals can be seen along bottom corners of image on either side. **b** Left PLHS: hippocampal striation seen better on the right side. On the left side, it is seen only in medial aspect of hippocampus, not in its lateral aspect. **c** Left PLHS: hippocampal striations seen on the right side, not on the left. **d** Right PLHS: hippocampal striation seen faintly on the left; not on the right. **e** Bilateral PLHS: hippocampal striation not well seen on either side

**Fig. 2:**
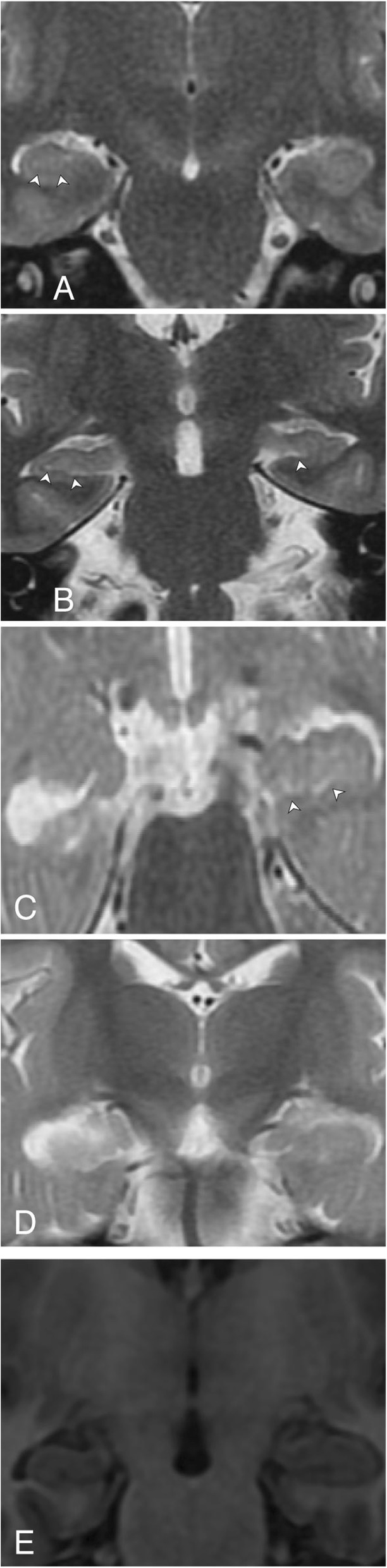
**a**–**d** High-resolution coronal T2-weighted images with magnification; **e** Coronal SPGR image with magnification. Arrowheads indicate hippocampal striation. **a** Left PLHS: hippocampal striation (arrowheads) seen better on the right side; not well made out on the left. Cochleae are seen symmetrical on both sides. **b** Left PLHS: hippocampal striation (arrowheads) seen better on the right side; seen only in medial aspect of the left hippocampus. Semicircular canals are seen symmetrical on both sides. **c** Right PLHS: hippocampal striation (arrowheads) seen on the left side; not on the right. The right hippocampus is also small, with increased signal intensity. **d** Bilateral PLHS: hippocampal striation not seen on either side; the right hippocampus is smaller in size. **e** Bilateral PLHS: small size of right hippocampus is also seen on coronal SPGR image

Between November 2010 and September 2011, we found 347 consecutive patients who had undergone our standard brain MR imaging epilepsy protocol. Children and adults with both generalised and focal seizures were imaged in this tertiary centre. Applying exclusion criteria: (a) patients not demonstrating PLHS and (b) patients who had undergone hippocampal surgery prior to this imaging, we had had 116 subjects demonstrating PLHS (Figs. 1 and 2).

Their age, sex, EEG findings (when available), side of PLHS (right, left, bilateral), presence/absence of hippocampal atrophy and hippocampal high intensity were recorded.

A small control population consisting of people who had no seizure history (imaged for other diseases) but had thin T2 coronal images (as part of protocol optimisation) were identified. In them, the following were recorded: age, sex, presence/absence of PLHS and indication for imaging.

### MR imaging

Studies were done at 1.5 T (Signa HDx, GE Healthcare, WI, USA) using a dedicated eight-channel phased array coil (Invivo Corporation, Gainesville, FL, USA). Oblique coronal T2-weighted sections were obtained perpendicular to the hippocampi (~ 20 slices) with the following parameters: TR 6000, TE 90, Matrix 384 × 256, FOV 22 × 16.5, NEX 2, slice thickness 3 mm with 0 interslice gap. The symmetry of semicircular canals (Fig. 1a and Fig. 2b) and cochleae (Fig. 2a) indicated that images were true coronal (with no obliquity of sections).

This high-resolution coronal T2 imaging took ~ 2 min which was clinically feasible. Other sequences like axial T1-W FLAIR, T2-W, T2-W FLAIR (slice thickness 5 mm; 1.5 mm interslice gap) and gradient echo (slice thickness 2 mm; − 1 mm interslice gap), coronal T2-weighted (slice thickness 5 mm; 2 mm interslice gap), sagittal T1/T2-weighted (slice thickness 5 mm; 1 mm interslice gap), and coronal three-dimensional fast spoiled gradient echo (slice thickness 2.2 mm; − 1.1 mm interslice gap) sequences were available. Coronal T2 FLAIR imaging was not done due to time constraints.

### Image interpretation

Visual assessment was done by experienced radiologists (AS (who had studied PLHS in 3 T) and SS) who were blinded to clinical and EEG findings; MRI findings were recorded by consensus. Positive PLHS was declared when more than two thirds of hippocampal striations were not seen in two or more consecutive T2 coronal images (Figs. 1 and 2) on motion-free images (when there was patient movement, the sequence had been repeated. In such cases, only the second set of motion-free images was used for image interpretation; so, the quality of images could be maintained). High signal intensity of the hippocampus was noted on coronal T2 images and the volume of the hippocampus was noted as normal or reduced (by eyeballing) on coronal spoiled gradient recalled echo (SPGR) sequence. Each MR image was displayed and interpreted on a high spatial resolution 1280 × 1024 monitor (Multisync 1990SXi, NEC, USA) with magnification (× 2).

### Statistical analysis

Age and sex distribution of subjects demonstrating PLHS were analysed using SPSS software. We did not attempt to calculate sensitivity and specificity of PLHS as definite gold standard could not be defined for hippocampal sclerosis (unlike previous studies [3, 5] that used clinical-electroencephalogram combination as a gold standard, our patients were referred by different clinicians and EEG correlation was not available in all patients).

## Results

The 116 PLHS subjects (age range 2–73 years, mean age 21.75, median age 16) included 62 males (53.9%; age range 2–68 years, mean age 21.6, median age 14.5) and 54 females (46.6%; age range 2–73 years, mean age 21.93, median age 16.5).

Sixty-six (56.9%) of our PLHS subjects were less than 18 years of age: 44 (37.9%) under the age of 12 years and 22 (19%) of 12–18 years of age.

Nineteen patients had definite extra-hippocampal pathology on routine MR imaging (seven sequelae of perinatal insult, five craniovertebral junction anomalies, two chronic infarcts, three gliotic, one sequela of meningoencephalitis, one arachnoid cyst).

Classic signs of hippocampal sclerosis (increased signal intensity and volume loss) were found in only 7 (6%) of the 116 subjects showing PLHS. All these 7 subjects also showed PLHS on the same side as the classic signs.

Awake EEG correlation was available in 91 (78.46%) of patients, of which 44 were positive. In one patient, positive EEG was obtained only during sleep EEG recording. Most common EEG abnormalities were purely frontal (18.2%) and purely temporal (13.2%); temporal lobe along with adjacent (parietal/frontal/occipital) lobes was focally involved in another 20%.

A 73-year-old woman who showed PLHS was clinically more likely to be ‘hippocampal sclerosis-associated with ageing’ [6] than Alzheimer’s disease [7]. The rest of the patients (115) were clinically ‘seizure disorder’.

Of the control population (25 subjects, age range 3–76 years, 17 males and 8 females), one showed PLHS. He was a primary CNS lymphoma patient who had completed treatment, with no history of seizures; EEG correlation was not available (we could not rule out absence seizures etc.). We feel that EEG correlation would be ideal in control population also, in future studies.

## Discussion

PLHS was much more common than hippocampal atrophy on MR imaging of epileptic patients. This finding in this study may be partially due to the milder cases of epilepsy in our patient population (refractory epilepsy—severe medial temporal lobe epilepsy (MTLE)— being considered for surgery were usually imaged in institutes focussing on epilepsy surgery; many cases referred to us were seizures well controlled with anti-epileptic drugs, where tapering/stopping medications was being considered).

We had initially wondered whether hippocampal striations and partial loss were seen in young children (youngest patients reported in previous studies were 14 [3] and 20 [5] years). But 44 (37.9%) of our PLHS subjects were under age of 12 years. We are not sure whether normal hippocampal striations would be seen in all normal children. Normal controls were difficult to find younger than 10 years (since children clinically referred for MRI at this age usually had some abnormality and many children (less than 7 years) require light/deep sedation due to noise and claustrophobia).

MRI and histopathological evidence of hippocampal sclerosis has been seen in children as young as 4 years [8]. Some studies have shown hippocampal abnormalities to be familial [9], and significant hippocampal asymmetry has been seen in asymptomatic relatives of hippocampal sclerosis patients [10], raising the possibility that such abnormal hippocampi may be epileptogenic when the child is febrile. The severity of hippocampal volume loss has been shown to correlate with both the number of secondary generalised seizures [11] and duration of epilepsy [12], suggesting that recurrent seizures cause further damage to the hippocampus.

We feel that PLHS is not specific for primary mesial temporal sclerosis (MTS), but can be seen in secondary MTS as well. The clinical and available EEG data point in this direction: even when EEG activity suggested other lobes (most common EEG abnormality (18.2%) was in the frontal lobe, followed by temporal lobe (13.6%)), PLHS was positive in the temporal lobes. Other lesions found on routine imaging (sequelae of perinatal insult, craniovertebral junction anomaly, etc.) may be the primary cause of seizures. Paucity of hippocampal digitations (a feature of MTLE, independent of hippocampal atrophy) observed in non-atrophic hippocampi contralateral to seizure foci at 7 T [5] also supports this postulation of secondary MTS. High (79%) frequency of dual pathology (cortical dysplasia along with hippocampal sclerosis) has been found in children and adolescents [8]. Studies showing hippocampal abnormalities in similar proportions in both acquired and developmental extra-hippocampal pathologies in children [13] imply that hippocampal abnormalities may be due to seizures from remote extra-hippocampal foci.

The finding of two hippocampal subregions ((1) lateral Ammon’s horn (CA1–CA3) and (2) dentate gyrus with CA4) being slightly smaller on left side of healthy controls at 7 T [5] may have some bearing on the fact that majority of our PLHS was on the left side.

Diagnosis of unilateral PLHS was relatively easy (due to comparison with the normal side), while that of bilateral PLHS needed strict application of the diagnostic criteria proposed by Hanamiya et al. [3]. This reiterates the need for validation of these diagnostic criteria.

Both ‘hippocampal sclerosis- associated with ageing’ [6] and Alzheimer’s disease [7] have hippocampal MRI changes. Unilateral/bilateral hippocampal atrophy and increased signal intensity have been described [14] in ‘hippocampal sclerosis dementia’ (another name for ‘hippocampal sclerosis-associated with ageing’). Adachi et al. [7] have demonstrated hypointense band along inner margin of hippocampus proper (corresponding to striations) on T2 and diffusion-weighted images at 1.5 T and noted that it is poorly visualised (corresponding to thinning of white matter in histopathology) in Alzheimer’s disease. They found diffusion-weighted images (DWI) better than T2-weighted images in identification of hippocampal striations, possibly due to the higher NEX and longer imaging time spent in DWI. Also, the very thick slices used (by Adachi et al.) for T2-weighted images may have affected the results.

Hanamiya et al. [3] speculated that hippocampal striations may correspond to stratum radiatum and/or stratum moleculare seen on ex vivo imaging [15], while others have called it superficial medullary lamina [7] and Ammon’s horn white matter [5]. This thin layer of white matter between the internal dentate gyrus and the external CA1, CA2, and CA3 subfields is a new radiographic landmark; it appears hypointense on the T2-weighted FSE acquisition and has been used in differentiating dentate/CA4 region from CA1–CA3 subfield in volumetry [16] studies. Howe et al. [17] recently demonstrated the T2 hypointense hippocampal layer on T2 FSTIR (fast inversion recovery) to be stratum lacunosum by 3 T MR scanning of temporal lobes (both in vivo (presurgical) and ex vivo (post-surgical specimens)) and correlating with histopathology. This is supported by ex vivo MR microscopy (correlating MR images with histological stains of the same sections) at 9.4 T [18] and 7 T [19] which found that very low signal intensity is seen in either heavily myelinated laminae (which may also contain iron) or highly cellular layers. MR microscopy (MRM) at 9.4 T [18] also found stratum lacunosum to be hypointense on MRM images with corresponding dip in signal intensity profile due to its high myelin content.

Howe et al. [17] showed that it is possible to see this T2 hypointense hippocampal layer on 1.5 T (though less clearly than on 3 T). Adachi et al. [7] also have demonstrated it on T2-weighted images at 1.5 T. We are demonstrating the hippocampal striations and their partial loss on T2 coronal images at 1.5 T. Periodically rotated overlapping parallel lines with enhanced reconstruction (PROPELLER) sequence which compensates for translational and rotational head movement during scan has been shown to be useful in demonstration of hippocampal striations at 3 T [20]. But to obtain coronal images perpendicular to the long axis of hippocampus, patient head had to be tilted forward (since we could not acquire the scans obliquely)—this forward tilting was not possible in some patients. Some people have found that they could get coronal PROPELLER images without bending the subjects’ neck.

Hippocampal volume loss (measured by manual or automatic methods—volumetry) is not sensitive or specific for hippocampal sclerosis. Studies have reported that up to 15% of hippocampal sclerosis patients may have normal hippocampal volumes [21] on MR imaging and that more than 10% difference between the right and left hippocampal volumes was a normal variation in some healthy people [22]. MR diffusion-weighted imaging [23] and MRS [24] were reported to be less sensitive than PLHS. Secondary MRI findings were reported to be more useful [25] than hippocampal volumes and MRS data. The temporopolar blurring of grey-white junction has been reported in MTS; examination of surgical specimens at 7 T showed axonal degeneration and significant decrease in the number of axons in those cases [26].

Cohen-Gadol et al. [27] had found patients whose clinical and EEG findings were consistent with MTLE, but no atrophy/increased signal intensity on MRI; they had termed them PTLE (paradoxical temporal lobe epilepsy). Cases showing only PLHS (and no classical signs of hippocampal sclerosis) may coincide with this PTLE.

Our high-resolution T2 imaging took ~ 2 min which was clinically feasible. Longer imaging time may increase the signal-to-noise ratio and hence the frequency of MR diagnosis.

Quantitative methods [3, 5, 16, 17] of detecting hippocampal sclerosis (by subfield volumetry—measuring thicknesses of CA1–3) consume time and effort. PLHS may be an easier technique for early detection of hippocampal sclerosis. PLHS may provide a surgical option in patients with refractory mesial epilepsy who have normal or nonspecific findings on routine MRI (labelled MR-negative TLE); recent reports show early surgery [28] to be very effective in pharmacoresistant epilepsy.

## Conclusion

Hippocampal striations have already been demonstrated at 1.5 T [7, 17], though called by different names (superficial medullary lamina [7] and hypointense band [17]). PLHS has been demonstrated and found to be useful in epilepsy on 3 T [3, 4] and on 7 T [5] MR. PLHS is demonstrated at 1.5 T in both adult and paediatric population in this article and is much more common than the classic signs of hippocampal sclerosis (increased signal intensity and volume loss).

Limitations of our study include lack of histological correlation (difficult to obtain in milder cases of hippocampal sclerosis where PLHS is likely more useful) or clinical gold standard (different referring clinicians). However, due to wider availability of 1.5 T (compared to 3 T) and significant clinical implications, we believe that studies with more rigorous criteria should be done to confirm the validity of (and lay down diagnostic criteria for) PLHS at 1.5 T.

## Data Availability

The datasets used and/or analysed during the current study are available from the corresponding author on reasonable request.

## Abbreviations

CA: Cornu ammonis
CNS: Central nervous system
DWI: Diffusion-weighted images
EEG: Electroencephalogram
FLAIR: Fluid-attenuated inversion recovery
FSTIR: Fast spin-echo short tau inversion recovery
MR: Magnetic resonance
MRI: Magnetic resonance imaging
MRM: Magnetic resonance microscopy
MRS: Magnetic resonance spectroscopy
MTLE: Medial temporal lobe epilepsy
MTS: Medial temporal sclerosis
NEX: Number of excitations
PET: Positron emission tomography
PLHS: Partial loss of hippocampal striation
PROPELLER: Periodically rotated overlapping parallel lines with enhanced reconstruction
SPECT: Single-photon emission computed tomography
SPGR: Spoiled gradient recalled echo
SPSS: Statistical Package for the Social Sciences
T: Tesla
TLE: Temporal lobe epilepsy
W: Weighted

## References

[1] Téllez-Zenteno José F., Dhar Raj, Wiebe Samuel (2005). Long-term seizure outcomes following epilepsy surgery: a systematic review and meta-analysis. Brain.

[2] McIntosh AM, Wilson SJ, Berkovic SF (2001). Seizure outcome after temporal lobectomy: current research practice and findings. Epilepsia.

[3] Hanamiya Mai, Korogi Yukunori, Kakeda Shingo, Ohnari Norihiro, Kamada Koji, Moriya Junji, Sato Toru, Kitajima Mika, Akamatsu Naoki, Tsuji Sadatoshi (2009). Partial Loss of Hippocampal Striation in Medial Temporal Lobe Epilepsy: Pilot Evaluation with High-Spatial-Resolution T2-weighted MR Imaging at 3.0 T. Radiology.

[4] Sen A, Chelladurai A, Emmanuel R (2009) 3 T MRI unit Bharat Scans Chennai India. Partial loss of hippocampal striation in 3 T imaging of mesial temporal lobe sclerosis. Poster presented at 12th Annual conference of Indian Society of NeuroRadiology, Chennai

[5] Henry TR, Chupin M, Lehéricy S et al (2011) Hippocampal sclerosis in temporal lobe epilepsy: findings at 7 T. Radiology 2011 261(1):199–209 10.1148/radiol.11101651 PMC3176424 21746814

[6] Nelson PT, Schmitt FA, Lin Y et al (2011) Hippocampal sclerosis in advanced age: clinical and pathological features. Brain 134:1506–1518 10.1093/brain/awr053 PMC3097889 21596774

[7] Adachi M, Kawakatsu S, Hosoya T (2003). Morphology of the inner structure of the hippocampal formation in Alzheimer disease. AJNR Am J Neuroradiol.

[8] Mohamed A., Wyllie E., Ruggieri P., Kotagal P., Babb T., Hilbig A., Wylie C., Ying Z., Staugaitis S., Najm I., Bulacio J., Foldvary N., Luders H., Bingaman W. (2001). Temporal lobe epilepsy due to hippocampal sclerosis in pediatric candidates for epilepsy surgery. Neurology.

[9] Kobayashi E, Lopes-Cendes I, Guerrerio CA, Sousa SC, Guerreiro MM, Cendes F (2001). Seizure outcome and hippocampal atrophy in familial mesial temporal lobe epilepsy. Neurology.

[10] Fernandez G., Effenberger O., Vinz B., Steinlein O., Elger C. E., Dohring W., Heinze H. J. (1998). Hippocampal malformation as a cause of familial febrile convulsions and subsequent hippocampal sclerosis. Neurology.

[11] Van Paesschen W, Connelly A, King MD, Jackson GD, Duncan JS (1997). The spectrum of hippocampal sclerosis: a quantitative magnetic resonance imaging study. Ann Neurol.

[12] Theodore W. H., Bhatia S., Hatta J., Fazilat S., DeCarli C., Bookheimer S. Y., Gaillard W. D. (1999). Hippocampal atrophy, epilepsy duration, and febrile seizures in patients with partial seizures. Neurology.

[13] Riney CJ, Harding B, Harkness WJF, Scott RC, Cross JH (2006). Hippocampal sclerosis in children with lesional epilepsy is influenced by age at seizure onset. Epilepsia.

[14] Mahieux F (2003) Sclérose hippocampique et démences. Psychol Neuropsychia 1:179–186 15683953

[15] WIESHMANN U. C., SYMMS M. R., MOTTERSHEAD J. P., MACMANUS D. G., BARKER G. J., TOFTS P. S., REVESZ T., STEVENS J. M., SHORVON S. D. (1999). Hippocampal layers on high resolution magnetic resonance images: real or imaginary?. Journal of Anatomy.

[16] McLean, John (2012) The investigation of hippocampal and hippocampal subfield volumetry, morphology and metabolites using 3 T MRI. PhD thesis, University of Glasgow. page 224( 8.5.7.5) > McLean J (2012) The investigation of hippocampal and hippocampal subfield volumetry, morphology and metabolites using 3 T MRI. PhD thesis, University of Glasgow. page 224

[17] Howe K.L., Dimitri D., Heyn C., Kiehl T.-R., Mikulis D., Valiante T. (2010). Histologically Confirmed Hippocampal Structural Features Revealed by 3T MR Imaging: Potential to Increase Diagnostic Specificity of Mesial Temporal Sclerosis. American Journal of Neuroradiology.

[18] Fatterpekar GM, Naidich TP, Delman BN (2002). Cytoarchitecture of the human cerebral cortex: MR microscopy of excised specimens at 9.4 Tesla. AJNR Am J Neuroradiol.

[19] Garbelli R., Zucca I., Milesi G., Mastropietro A., D'Incerti L., Tassi L., Colombo N., Marras C., Villani F., Minati L., Spreafico R. (2011). Combined 7-T MRI and histopathologic study of normal and dysplastic samples from patients with TLE. Neurology.

[20] Eriksson SH, Thom M, Bartlett PA (2008). PROPELLER MRI visualizes detailed pathology of hippocampal sclerosis. Epilepsia.

[21] Jackson GD, Kuzniecky RI, Cascino GD (1994). Hippocampal sclerosis without detectable hippocampal atrophy. Neurology.

[22] Cheon JE, Chang KH, Kim HD (1998). MR of hippocampal sclerosis: comparison of qualitative and quantitative assessments. AJNR Am J Neuroradiol.

[23] Wehner T, Lapresto E, Tkach J (2007). The value of interictal diffusion-weighted imaging in lateralizing temporal lobe epilepsy. Neurology.

[24] Capizzano AA, Vermathen P, Laxer KD (2002). Multisection proton MR spectroscopy for mesial temporal lobe epilepsy. AJNR Am J Neuroradiol.

[25] Lopez-Acevedo ML, Martinez-Lopez M, Favila R, Roldan-Valadez E (2012). Secondary MRI-findings, volumetric and spectroscopic measurements in mesial temporal sclerosis. Swiss Med Wkly..

[26] Garbelli R., Milesi G., Medici V., Villani F., Didato G., Deleo F., D'Incerti L., Morbin M., Mazzoleni G., Giovagnoli A. R., Parente A., Zucca I., Mastropietro A., Spreafico R. (2012). Blurring in patients with temporal lobe epilepsy: clinical, high-field imaging and ultrastructural study. Brain.

[27] Cohen-Gadol AA, Bradley CC, Williamson A (2005). Normal magnetic resonance imaging and medial temporal lobe epilepsy: the clinical syndrome of paradoxical temporal lobe epilepsy. J Neurosurg.

[28] Engel Jerome (2012). Early Surgical Therapy for Drug-Resistant Temporal Lobe Epilepsy. JAMA.

